# The Depression in Visual Impairment Trial (DEPVIT): trial design and protocol

**DOI:** 10.1186/1471-244X-12-57

**Published:** 2012-07-12

**Authors:** Tom H Margrain, Claire Nollett, Julia Shearn, Miles Stanford, Rhiannon Tudor Edwards, Barbara Ryan, Catey Bunce, Robin Casten, Mark T Hegel, Daniel J Smith

**Affiliations:** 1 School of Optometry and Vision Sciences, Cardiff University, Cardiff, CF24 4LU, UK; 2 Institute of Health and Wellbeing, College of Medical, Veterinary and Life Sciences, University of Glasgow, Gartnavel Royal Hospital, 1055 Great Western Road, Glasgow, G12 0XH, UK; 3 Eye (Ophthalmology) team, South Wing, St Thomas' Hospital, Westminster Bridge Road, London, SE1 7EH, UK; 4 Centre for Economics and Policy in Health/Canolfan Economeg a Pholisi Iechyd IMSCaR, College of Health and Behavioural Sciences, Bangor University, Dean Street Building, Gwynedd, LL57 1UT, UK; 5 Moorfields Eye Hospital, City Road, London, EC1V 2PD, UK; 6 Department of Psychiatry and Human Behaviour, Jefferson Medical College, Philadelphia, USA; 7 Department of Psychiatry, Dartmouth Medical School, Hanover, NH, USA

## Abstract

**Background:**

The prevalence of depression in people with a visual disability is high but screening for depression and referral for treatment is not yet an integral part of visual rehabilitation service provision. One reason for this may be that there is no good evidence about the effectiveness of treatments in this patient group. This study is the first to evaluate the effect of depression treatments on people with a visual impairment and co morbid depression.

**Methods /design:**

The study is an exploratory, multicentre, individually randomised waiting list controlled trial. Participants will be randomised to receive Problem Solving Therapy (PST), a ‘referral to the GP’ requesting treatment according to the NICE’s ‘stepped care’ recommendations or the waiting list arm of the trial. The primary outcome measure is change (from randomisation) in depressive symptoms as measured by the Beck’s Depression Inventory (BDI-II) at 6 months. Secondary outcomes include change in depressive symptoms at 3 months, change in visual function as measured with the near vision subscale of the VFQ-48 and 7 item NEI-VFQ at 3 and 6 months, change in generic health related quality of life (EQ5D), the costs associated with PST, estimates of incremental cost effectiveness, and recruitment rate estimation.

**Discussion:**

Depression is prevalent in people with disabling visual impairment. This exploratory study will establish depression screening and referral for treatment in visual rehabilitation clinics in the UK. It will be the first to explore the efficacy of PST and the effectiveness of NICE’s ‘stepped care’ approach to the treatment of depression in people with a visual impairment.

**Trial registration:**

ISRCTN46824140

## Background

The presence of a visual impairment is known to increase the risk of depression and it is an independent predictor of suicide in the elderly [1,2]. Epidemiological studies indicate that the prevalence of depression in visually impaired older adults living in the UK is 13.5% compared to just 7.4% for those without a visual impairment [3]. At the time visually impaired people most need help, when they are accessing vision rehabilitation services, data from North America indicates that the prevalence of depression is higher still at about 30% [4]‐[7].

Low vision rehabilitation services operate at the interface between health and social care and, in the UK, typically involve optometrists working with social services and the voluntary sector. The aim of this intervention is to promote independent [8] living by reducing the disability associated with visual impairment. More specifically, people with low vision are provided with practical solutions to specific problems e.g. by providing magnifiers to help with reading, improved lighting and modifications to the home [9]. However, although these services have been shown to reduce disability and people appreciate the low vision aids provided, their reported effects on overall quality-of-life have been modest [10]‐[12]. One contributory factor is likely to be untreated depression in this patient group. Depression is not only disabling in its own right but is very likely to act as a barrier to good vision rehabilitation outcomes [13,14].

Despite the fact that people with a visual impairment have an increased risk of depression, depression screening and referral for treatment is not currently part of vision rehabilitation service provision. One reason for this apparent omission may be that there is no good evidence about the effectiveness of depression treatments in this specific patient group. For example, although the National Institute for Health and Clinical Excellence (NICE) have issued guidance on the management and treatment of depression in people with chronic health problems there is no evidence about the effectiveness of the recommended ‘stepped care’ approach in people with a visual impairment [15]. The ‘stepped care’ treatment options include: physical activity programmes, group-based peer support (self-help) programmes, individual guided self-help programmes based on the principles of CBT including behavioural activation and Problem Solving Therapy (PST) and, antidepressants. Although there is no direct evidence about the effectiveness of these interventions in people with depression and a visual impairment, the ability of PST to prevent the onset of depression in people with age-related macular degeneration, the leading cause of visual impairment in the developed world, has been studied [8]. In that study PST was shown to be successful at 2 months but the effects were less impressive at 6 months.

The Depression in Visual Impairment Trial (DEPVIT) will establish depression screening in several UK based vision rehabilitation centres and obtain preliminary data about the treatment effects associated with the stepped care approach recommended by NICE and PST. More specifically, DEPVIT aims to estimate the prevalence of depressive symptoms using the Geriatric Depression Scale (GDS-15) in an observational study of consecutive attendees of low vision services in England and Wales, and compare 2 interventions, PST and ‘referral to the GP’, against a waiting list control for newly-diagnosed low vision patients with depressive symptoms in an exploratory randomised controlled trial. Subsidiary aims include determining what action GPs take in response to a referral for depressive symptoms in this patient group, determining the acceptability of these interventions to patients and establishing economic information about the cost to roll-out a psychological problem solving based intervention for people experiencing sight loss and how well standard economic tools work with people experiencing sight loss.

## Methods/design

**Figure 1:**
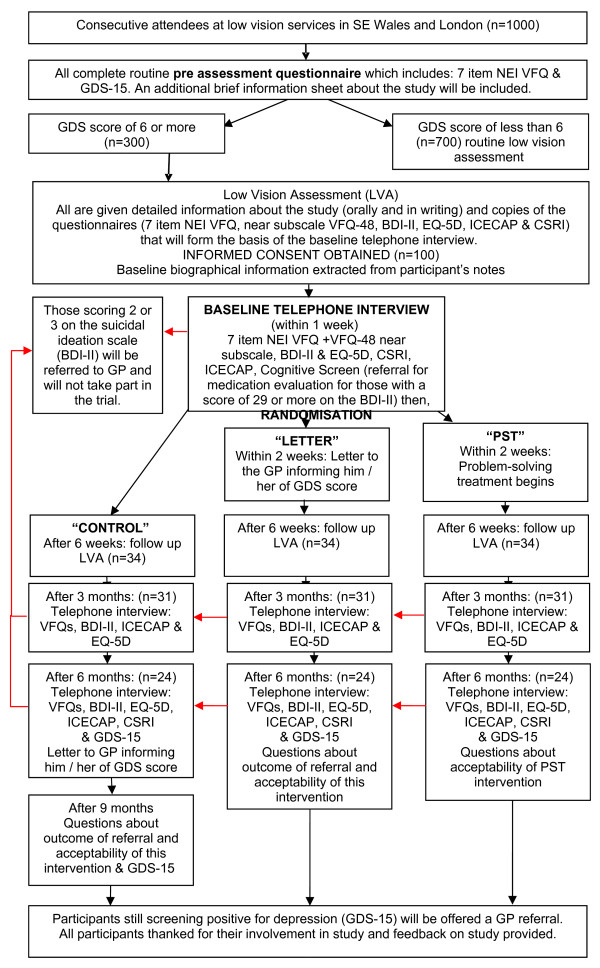
DEPVIT Trial Design.

This is an exploratory, multicentre, individually randomised controlled trial. Figure 1 summarises the study design and the flow of patients through the trial.

### The interventions

This trial involves 2 active interventions PST and ‘referral to the GP’ and a ‘waiting list control’.

The PST intervention evaluated in this study is a behavioural therapy that has been specifically designed for people with a visual impairment [8]. This intervention involves a trained psychological therapist working with the patient in their own home or at one of the research centres. Over a 6–8 week period, patients are taught a 7-step method for approaching and solving their problems. The steps are: (1) defining the problem; (2) establishing realistic goals; (3) brainstorming solutions; (4) implementing decision making guidelines (Pros versus Cons); (5) choosing a preferred solution; (6) implementing the solution; and (7) evaluating the outcome. The PST intervention will also include additional self-help materials for patients e.g. materials concerning an explanation of depression, a discussion on the importance of treating depression, and a description of the various treatment options as well as vision related sign posting materials. This type of behavioural therapy is an individualised guided self help approach consistent with NICE guidelines [15]. Optometrists providing the low vision assessment will share the patient’s ‘treatment plan’ with the therapist (via a brief report) so that the therapist can help the patient implement the optometrist’s recommendations via the PST framework if this is what the patient wishes. To ensure the fidelity of this intervention the PST therapists will be trained by Dr Mark Hegel the Clinical Psychologist who originally designed the PST intervention used in 2007 by Rovner, Casten et al, [8]. With the participants consent, each PST intervention will be taped and one third of randomly selected tape recordings will be reviewed to ensure the fidelity of the intervention.

The second intervention is ‘referral to the GP’. A carefully crafted referral letter will inform the GP that their visually impaired patient has screened positive for a possible depressive disorder and invites them to offer the patient an assessment and treatment as per NICE guidelines for the management of depression. This is a pragmatic intervention and we acknowledge that GPs may or may not adhere to NICE guidelines i.e. we propose to evaluate the effectiveness of the referral, not ‘best practice’ as described by NICE. The fidelity of this intervention will be assessed by recording the number of correctly addressed referral letters sent.

The final arm of the study is a ‘waiting list control’. Patients in this arm of the trial will be referred to their GP using the referral letter described above after the 6 month outcome measure. A ‘waiting list control’ provides people taking part in the study with a better level of care than ‘treatment as usual’ because depressive symptoms are currently ignored and there is no referral. The fidelity of this intervention will be assessed by asking people at 6 months about any treatments received during the time of the study.

In addition to the interventions listed above, all participants in the study will have a follow up vision rehabilitation appointment 6 weeks after the initial appointment. Follow up appointments of this type are typical for people who appear depressed or are obviously struggling at the initial low vision assessment.

### Outcome measures

The primary outcome will be the change in depressive symptoms, from randomisation to 6 months, as measured by the Becks Depression Inventory (BDI-II). The BDI-II is a reliable, valid and widely used outcome measure for depression [16,17].

Secondary outcome measures in this exploratory trial will include change (from randomisation to 3 and 6 months) in visual disability as measured using the 7 item NEI VFQ and the near vision subscale of the VFQ-48 and, change in ‘generic health related quality of life’ as measured using the EQ-5D [18,19]. Additional secondary outcomes measured at 6 months will include the proportion of people still screening positive for depression as measured with the GDS-15, participant’s use of services as determined using a Client Services Receipt Inventory (CSRI) and an economic measure of ‘capability and wellbeing’ the ICECAP-O [20]‐[22]. At the end of this exploratory trial participants will be asked about any treatments for depression they have received during the trial period (specifically, whether they received regular review and support by their GP, referral to a community mental health team, supportive psychotherapy, counselling, problem-solving therapy, cognitive-behavioural therapy or antidepressant therapy). They will also be asked about the acceptability of any interventions received. Participants in the waiting list control group who still screen positive for depression (GDS-15, 6+) will be referred to their GP. And, 3 months later asked about any treatment for depression received as a result of the referral.

All trial outcomes will be assessed by telephone interview by the Study Coordinator who will be masked to the treatment intervention. In order to minimise ‘loss to follow up’, interviews will be based on a standard script and will remind participants of their valuable contribution and the need to complete the study.

### Sample size

During the course of the study 1000 consecutive attendees will be screened. At the outset we anticipate that about 1/3 will screen positive for depression and be eligible, and that of those, about 100 will consent to take part. This target of 100 patients recruited into the trial is realistic and achievable. Resource limitations require us to cap the number of participants to 150.

Since this study is exploratory we will predominantly use descriptive methods to report results. That is, this study is not powered to look at differences in outcomes (there is no data upon which to determine the sample size). However, to give the reader some indication about the power of this exploratory trial, if we assume that those entering the trial are moderately depressed (average BDI-II score of 30, SD 10.5) and, if the trial finishes with only 20 participants per treatment group, we would have 83% power to detect a difference in post treatment means of 10 (a change of 10 units corresponding to a moderately important clinical difference) with a 5% two-sided significance level.

### Recruitment

Participants will be identified and recruited from 7 community based low vision rehabilitation centres in South Wales and from one large secondary care based low vision rehabilitation centre at Guys and St Thomas’ NHS Trust, London [23]. Based on known activity in these recruitment centres we anticipate that the 7 sites in Wales will screen approximately 500 people in total and that a similar number will be screened in London.

### Inclusion criteria

Adults (18+ years of age) with a score of at least 6 on the GDS-15 (administered as part of the routine pre assessment questionnaire) will be the inclusion criterion for this study.

### Exclusion criteria

To ensure our results are applicable to the population we propose to keep the exclusion criteria to a minimum, specifically: 1) those who have already had a low vision assessment within the previous 12 months, 2) those who are referred to the clinic in error (i.e. so visually impaired that low vision care and follow up are inappropriate or, so visually able (e.g. following refraction) that there are no low vision needs), 3) those already being treated for depression including psychotherapeutic and psychopharmacological treatments, 4) inability to understand English, 5) Inability to use the telephone e.g. caused by very poor hearing, 6) severe medical illness that would preclude participation in a 6 month study, 7) a score of 2 or 3 on the BDI-II question about suicidal ideation, all people in this group will be urgently referred to their GP, 8) those screening positive for significant cognitive / memory problems as determined with a modified version of the MMSE will be excluded.

### Trial procedures: consent, baseline assessments and randomisation

The low vision rehabilitation services in Wales and London now include a routine self-complete pre assessment questionnaire (made up of the 7 item NEI VFQ and GDS-15) which is given to patients one week before their consultation. During the trial this will be accompanied by a Patient Information Sheet letting people know that the low vision service they are about to attend is taking part in a study. The sheet details the aims, purpose and what is involved in participation in the study. At the rehabilitation consultation, those satisfying the inclusion criteria will be invited to take part by the optometrist who provides the low vision rehabilitation intervention.

The optometrists (who are trained to take informed consent) will document the consultation using standard clinical records and, for the purposes of this study, a case report form. The GDS-15 will be reviewed at the start of the hour long low vision assessment and all positive screening patients will be informed that they may be eligible to take part in the study. Toward the end of the low vision assessment a full verbal explanation of the study will be provided to positive screening patients and they will be asked if they would like to take part. Those expressing an interest in taking part will then be screened by the optometrist for exclusion criteria 1, 2, 3, 4, 5 & 6. Those still eligible will be given written copies (large print) of the questionnaires that will be used in the telephone interview to take home and will be asked to provide their written informed consent.

Consenting patients will be contacted by the Study Coordinator within 1 week to, make an appointment to administer the cognitive screen and BDI-II to check for the exclusion criteria 7 and 8. Those meeting the additional eligibility criteria will be enrolled in the study and the other baseline outcome measures will be administered. Those scoring 29 or more on the BDI-II at the baseline interview have severe depressive symptoms and in all cases a letter will be written to the person’s GP requesting a ‘medication evaluation’.

Potential recruits who would like additional time to consider enrolment, after the initial low vision assessment will be contacted by phone by their low vision practitioner one week later to find out “how they are getting on”. If they then want to take part in the study they will be booked back in to see the practitioner to complete the study consent form. Participants who screen positive for depression but who decline to take part in the trial will be asked if they would like to be referred to their GP anyway. The person’s decision will be noted in the records e.g. if they decline referral. These people will be asked if they are willing to provide their consent for the use of anonymous baseline data.

Within one week of the baseline telephone interview, participants will be randomised using computer generated permuted blocks of varying sizes (unknown to investigators) stratified for study centre (Wales and London) and for ‘medication evaluation’ only. To ensure that the Study Coordinator is masked to treatment allocation, the Chief Investigator will allocate participants to the different arms of the trial.

For those assigned to the ‘referral to GP’ arm of the trial, the letter to the GP will be posted on the day the person was randomised and the participant will be asked to make an appointment with their GP within 2 weeks. For those assigned to PST, treatment will commence within 2 weeks of randomisation. All participants will be given a follow up vision rehabilitation appointment 6 weeks after the initial rehabilitation appointment.

The Study Coordinator will contact participants 3 and 6 months after randomisation to obtain outcome measure data.

### Process evaluation

A process evaluation will be conducted to evaluate whether the intervention was carried out in accordance with the trial protocol. Specifically, all PST consultations will be taped and 1/3 of them will be reviewed by the research team to ensure the fidelity of this intervention. The fidelity of the ‘referral to GP’ arm will be determined by noting the date the referral letter was sent to the GP. After the 6 month outcome measure a letter will be sent to each participant’s GP asking what treatments / action have taken place during the trial period. At the end of the trial all participants will take part in an interview to determine their level of satisfaction with the service received and any action taken by the GP. This final interview will include qualitative questions about what worked well and not so well.

### Assessment of costs

We will also conduct an assessment of the direct costs associated with the delivery of the PST intervention and identify its key cost consequences e.g. reductions in demands on other service. The cost of the intervention will be assessed by monitoring the activity of the Psychological Therapist and the costs associated with travel to and from participants’ homes.

The costs associated with the use of additional services will be established using a telephone based Client Service Receipt Inventory (CSRI) [21].

### Analyses

#### Statistical analyses

We will tabulate the number of patients by GDS-15 Score. We will estimate the prevalence of depressive symptoms (defined as a GDS-15 score of 6 or more) with 95% confidence intervals computed by the exact binomial method. We will use logistic regression to assess whether factors such as age, sex, pathology and site are associated with depressive symptoms. With a sample size of 1000, a two sided 95% confidence interval will extend 0.028 from the observed proportion for an expected proportion of 0.3.

This trial is the first of its kind and hence ‘exploratory’. For the trial, we will compare baseline characteristics of the allocated groups to ensure the adequacy of randomisation (including duration of visual loss). If outcome data are not available for all randomised patients, we will compare characteristics of those for whom it is available with those for whom it is not to assess whether the groups differ systematically. To retain the validity of the randomisation process the analyses will be undertaken on an intention-to-treat basis.

We will report average change in BDI scores in treatment groups using the mean or median as appropriate together with some indication of variability such as the standard deviation or interquartile range. These results will then be used to estimate the numbers of patients required for a definitive study.

Missing data has the potential to bias our findings. Hence we will attempt to minimise ‘loss to follow up’ by making it clear to potential recruits what is required at the outset, letting them know that completion of the study is important and reminding them of the value of their contribution when outcomes are assessed during the scripted telephone interviews. We will document the reasons for missing data and establish both the extent and patterns of missing data in each arm of the trial. We will also acknowledge the potential impact of missing data in our conclusions.

We will also compare the characteristics (age, sex, level of depression, level of vision impairment, ethnicity, functional ability and time since vision loss first identified) of those who did and did not consent to take part in the trial to determine if we can predict who is likely to benefit from depression screening.

We will also look for PST trainer effects (i.e. compare outcomes of PST delivery in London and Wales). The study design and scripted outcome measurements are designed to minimise the possibility of masking violation in this exploratory trial. We will however report any masking violations descriptively.

### Analyses of costs

We will determine the direct costs of the psychological problem solving based intervention for people experiencing sight loss. We will also determine preliminary data on the incremental cost effectiveness of a home-based problem solving psychological intervention, aimed to support people experiencing sight loss, compared to referral to primary care and a waiting list control.

From a public sector, multi-agency perspective we will: fully cost the development, staff training and roll-out of this problem-solving psychological intervention [24]‐[26]. We will record study participant primary and secondary care health service use, social care use and contacts with voluntary sector agencies supporting blind and partially sighted people (using an interviewer administered, telephone-based Client Service Receipt Inventory (CSRI), as one of the full battery of study measures, costed using National unit costs and where necessary, local costs). We will also conduct a primary cost effectiveness analysis (using the Geriatric Depression Scale (GDS-15) as our measure of effectiveness); conduct a secondary cost-utility analysis using EQ-5D (interviewer administered by telephone) as our measure of utility to generate a cost per QALY (ICER) and Cost Effectiveness Acceptability Curve (CEAC) for comparison with the NICE ceiling of £30,000 [25,27].

As this is a feasibility study we will pay particular attention to the validity of EQ-5D in this group of patients, comparing the 5 domains of EQ-5D (including anxiety and depression) with, in particular, the depression measures used in this study [28].

## Discussion

This trial will be the first to evaluate the effectiveness and acceptability of depression treatments in people with a visual impairment. This study is significant because about 155,000 visual rehabilitation appointments are provided for people with a visual impairment in the UK each year and about 1/3 of them are likely to have significant depressive symptoms [29]. The trial will establish depression screening and referral for treatment in this high risk group.

This exploratory trial will produce data on the effects of PST and ‘referral to the GP’ on depressive symptoms, visual function, generic health related quality-of-life and use of services. Together with the economic evaluation and information about the acceptability of these interventions the data from this trial will inform the design of a larger (phase III) randomised controlled trial.

### Strengths of the study

To maximise the potential impact of PST, the research team includes the original developers of this manualised intervention. The PST intervention used in this study draws on the significant experience of the research team who have worked with people with a visual impairment and depression for many years.

The ‘referral to GP’ arm of the trial is a pragmatic intervention designed to find out what really happens in response to a referral for depression. Although our referral letter encourages GPs to offer treatment according to current NICE guidelines it is the effectiveness of the referral we are evaluating not the ‘stepped care approach’ recommended by NICE. That is, we do not expect all GPs to adhere to NICE guidelines. By contacting GPs at the end of the trial and by speaking to participants randomised to this arm of the study we should gain a clear understanding of what actually happens following a referral.

The study materials including the Patient Information Sheet and Consent Form have been developed in collaboration with service users. The pre assessment questionnaire that includes the GDS-15 and the 7 item NEI VFQ has been piloted extensively on people with a visual impairment, including those with depression.

In line with MRC guidelines on good clinical practice in clinical trials we have established an independent Trial Steering Committee which will ensure that the protocol is adhered to.

### Challenges

Encouraging those with depression to engage with the research project may be challenging given that their motivation is likely to be low. By getting their optometrist, who has provided the vision rehabilitation intervention, to describe the study and ask for consent we hope to maximise recruitment.

The relationship between depression and visual impairment is complex [13]. Hence, good outcomes are most likely to be observed following good vision rehabilitation and depression interventions i.e. both are required. Whilst the research team have good control of the depression interventions and will check their fidelity we are not checking the rigor of the vision rehabilitation interventions. The optometrists providing the vision rehabilitation in Wales have been trained and accredited, by Cardiff University, to provide a very specific type of community based intervention delivered as part of the “Welsh Low Vision Service” [23,30]. The optometrists at the London site are experienced vision rehabilitation practitioners but they have not been trained and accredited in the same way. To minimise any differences in the vision rehabilitation intervention the clinical leads of the Welsh and London services have met to agree to a standardised approach to low vision rehabilitation. This standardised approach is documented in the master files at all sites.

## Conclusion

In summary, although recent evidence suggests that depression is likely to be prevalent in people attending vision rehabilitation services, screening for depression and referral for treatment is not currently part of service. This study will for the first time establish depression screening and referral for treatment in UK based vision rehabilitation clinics. It will provide preliminary data on the effects of ‘Problem Solving Therapy’ and a ‘referral to the GP’ on depressive symptoms and visual function in people with untreatable visual impairment. Information about the effectiveness of depression treatments in this group will help shape future service delivery.

## Ethical and governance approvals

This study has been approved by the South East Wales Research Ethics Committee Panel - C (11/WA/0014).

## Competing interests

The authors declare that they have no competing interests.

## Authors’ contributions

THM and DS drafted this paper which was added to and modified by all other authors. The PST intervention was developed by MH. All authors contributed to the design of the study protocol. All authors read and approved the final manuscript.

## Pre-publication history

The pre-publication history for this paper can be accessed here:

http://www.biomedcentral.com/1471-244X/12/57/prepub

## References

[B1] Osborn DPJ Fletcher AE Smeeth L Stirling S Bulpitt CJ Breeze E Ng ESW Nunes M Jones D Tulloch A Factors associated with depression in a representative sample of 14 217 people aged 75 and over in the United Kingdom: results from the MRC trial of assessment and management of older people in the community Int J Geriatr Psychiatry 2003 18 7 623 630 10.1002/gps.896 12833307

[B2] Waern M Rubenowitz E Runeson B Skoog I Wilhelmson K Allebeck P Burden of illness and suicide in elderly people: case–control study Br Med J 2002 324 7350 1355 1357 10.1136/bmj.324.7350.1355 12052799 PMC115206

[B3] Evans JR Fletcher AE Wormald RPL Depression and anxiety in visually impaired older people Ophthalmology 2007 114 2 283 288 10.1016/j.ophtha.2006.10.006 17270678

[B4] Brody BL Gamst AC Williams RA Smith AR Lau PW Dolnak D Rapaport MH Kaplan RM Brown SI Depression, visual acuity, comorbidity, and disability associated with age-related macular degeneration Ophthalmology 2001 108 10 1893 1900 10.1016/S0161-6420(01)00754-0 11581068

[B5] Brody BL Roch-Levecq AC Thomas RG Kaplan RM Brown SI Self-management of age-related macular degeneration at the 6-month follow-up - A randomized controlled trial Arch Ophthalmol 2005 123 1 46 53 10.1001/archopht.123.1.46 15642811

[B6] Rovner BW Casten RJ Neuroticism predicts depression and disability in age-related macular degeneration J Am Geriatr Soc 2001 49 8 1097 1100 10.1046/j.1532-5415.2001.49215.x 11555073

[B7] Rovner BW ShmuelyDulitzki Y Screening for depression in low-vision elderly Int J Geriatr Psychiatry 1997 12 9 955 959 10.1002/(SICI)1099-1166(199709)12:9<955::AID-GPS672>3.0.CO;2-5 9309476

[B8] Rovner BW Casten RJ Hegel MT Leiby BE Tasman WS Preventing depression in age-related macular degeneration Arch Gen Psychiatry 2007 64 8 886 892 10.1001/archpsyc.64.8.886 17679633

[B9] Margrain TH Helping blind and partially sighted people to read: the effectiveness of low vision aids Br J Ophthalmol 2000 84 8 919 921 10.1136/bjo.84.8.919 10906105 PMC1723574

[B10] Harper R Doorduyn K Reeves B Slater L Evaluating the outcomes of low vision rehabilitation Ophthalmic Physiol Opt 1999 19 1 3 11 10.1046/j.1475-1313.1999.00411.x 10615433

[B11] Scott IU Smiddy WE Schiffman J Feuer WJ Pappas CJ Quality of life of low-vision patients and the impact of low-vision services Am J Ophthalmol 1999 128 1 54 62 10.1016/S0002-9394(99)00108-7 10482094

[B12] Stelmack JA Stelmack TR Massof RW Measuring low-vision rehabilitation outcomes with the NEI VFQ-25 Invest Ophthalmol Vis Sci 2002 43 9 2859 2868 12202503

[B13] Horowitz A Reinhardt JP Boerner K The effect of rehabilitation on depression among visually disabled older adults Aging Ment Health 2005 9 6 563 570 10.1080/13607860500193500 16214704

[B14] Murray CJL Lopez AD Alternative projections of mortality and disability by cause 1990–2020: Global burden of disease study Lancet 1997 349 9064 1498 1504 10.1016/S0140-6736(96)07492-2 9167458

[B15] NICE Depression in adults with a chronic physical health problem: Treatment and management 2009 National Institute for Health and Clinical Excellence, London

[B16] Beck AT Erbaugh J Ward CH Mock J Mendelsohn M An inventory for measuring depression Arch Gen Psychiatry 1961 4 6 561 10.1001/archpsyc.1961.01710120031004 13688369

[B17] Beck AT Steer RA Garbin MG Psychometric properties of the Beck Depression Inventory inventory - 25 years of evaluation Clin Psychol Rev 1988 8 1 77 100 10.1016/0272-7358(88)90050-5

[B18] Rabin R de Charro F EQ-5D: a measure of health status from the EuroQol Group Ann Med 2001 33 5 337 343 10.3109/07853890109002087 11491192

[B19] Ryan B Court H Margrain TH Measuring low vision service outcomes: Rasch analysis of the seven-item National Eye Institute Visual Function Questionnaire Optom Vis Sci 2008 85 2 112 121 10.1097/OPX.0b013e31816225dc 18296928

[B20] Almeida OP Almeida SA Short versions of the geriatric depression scale: A study of their validity for the diagnosis of a major depressive episode according to ICD-10 and DSM-IV Int J Geriatr Psychiatry 1999 14 10 858 865 10.1002/(SICI)1099-1166(199910)14:10<858::AID-GPS35>3.0.CO;2-8 10521885

[B21] Chisholm D Knapp MRJ Knudsen HC Amaddeo F Gaite L van Wijngaarden B Grp ES Client Socio-Demographic and Service Receipt Inventory - European Version: Development of an instrument for international research - EPSILON Study 5 Br J Psychiatry 2000 177 S28 S33 10.1192/bjp.177.39.s28 10945075

[B22] Coast J Peters TJ Natarajan L Sproston K Flynn T An assessment of the construct validity of the descriptive system for the ICECAP capability measure for older people Qual Life Res 2008 17 7 967 976 10.1007/s11136-008-9372-z 18622721

[B23] Margrain TH Ryan B Wild JM A revolution in Welsh low vision service provision Br J Ophthalmol 2005 89 8 933 934 10.1136/bjo.2005.066142 16024836 PMC1772790

[B24] Council MR A framework for development and evaluation of RCT’s for complex interventions to improve health 2008 Medical Research Council, London

[B25] Edwards RT Ceilleachair A Bywater T Hughes DA Hutchings J Parenting programme for parents of children at risk of developing conduct disorder: cost effectiveness analysis Br Med J 2007 334 7595 682 685 10.1136/bmj.39126.699421.55 17350965 PMC1839236

[B26] Edwards RT Hounsome B Linck P Russell IT Economic evaluation alongside pragmatic randomised trials: developing a standard operating procedure for clinical trials units Trials 2008 9 64 10.1186/1745-6215-9-64 19014634 PMC2615739

[B27] Fenwick E O’Brien BJ Briggs A Cost-effectiveness acceptability curves - facts, fallacies and frequently asked questions Health Econ 2004 13 5 405 415 10.1002/hec.903 15127421

[B28] Brazier JE Yang Y Tsuchiya A Rowen DL A review of studies mapping (or cross walking) non-preference based measures of health to generic preference-based measures Eur J Health Econ 2010 11 2 215 225 10.1007/s10198-009-0168-z 19585162

[B29] Culham LE Ryan B Jackson AJ Hill AR Jones B Miles C Young JA Bunce C Bird AC Low vision services for vision rehabilitation in the United Kingdom Br J Ophthalmol 2002 86 7 743 747 10.1136/bjo.86.7.743 12084742 PMC1771185

[B30] Ryan B White S Wild J Court H Margrain TH The newly established primary care based Welsh Low Vision Service is effective and has improved access to low vision services in Wales Ophthalmic Physiol Opt 2010 30 4 358 364 10.1111/j.1475-1313.2010.00729.x 20492541

